# Surgical tagging of Atlantic mackerel (*Scomber scombrus*): electroanaesthesia and survival in captivity and the field

**DOI:** 10.1111/jfb.70317

**Published:** 2026-01-22

**Authors:** Caliyena R. Brown, Morgan L. Piczak, Elisabeth Van Beveren, John Batt, Robert J. Lennox

**Affiliations:** ^1^ Department of Biology Dalhousie University Halifax Nova Scotia Canada; ^2^ Fisheries and Oceans Canada, Institut Maurice‐Lamontagne Mont‐Joli Quebec Canada; ^3^ Aquatron Laboratory Dalhousie University, Life Sciences Centre Halifax Nova Scotia Canada; ^4^ Ocean Tracking Network, Steele Ocean Science Building Halifax Nova Scotia Canada

**Keywords:** acoustic tagging, biotelemetry, fish handling, surgical tagging, forage fish, movement ecology, survival analyses

## Abstract

Electroanaesthesia using electric fish handling gloves induced rapid and reversible sedation of Atlantic mackerel (*Scomber scombrus*) during surgical tagging without observable adverse effects on behaviour or tag retention. In the laboratory, survival analyses revealed that longer handling times and smaller body sizes significantly reduced survival rates. Particularly, most mortalities were associated with visible skin lesions and signs of infection, likely resulting from initial capture, transport stress and sensitivity to captivity. Wound assessments showed that surviving mackerel generally exhibited fully healed or minorly red incision sites, indicative of promising wound healing post‐surgical tagging. Field trials using refined methods, including minimized handling and optimal capture methods, yielded a 92% survival rate, with only 4 of 50 fish classified as post‐tagging mortalities based on acoustic telemetry detections. Statistical analyses confirmed significantly higher survival rates in the field compared to the laboratory. Outcomes from both settings informed the establishment of best tagging practices for Atlantic mackerel, providing a framework for improving fish welfare and enhancing the reliability of biotelemetry studies for this species.

## 1 INTRODUCTION

The Atlantic mackerel (*Scomber scombrus*, herein mackerel) is a highly migratory pelagic forage fish widely distributed throughout the North Atlantic. As a link between zooplankton and higher trophic levels (Savenkoff et al., 2005), mackerel play a key ecological role as an energy‐rich food source for several predators across diverse taxa (Moustahfid et al., 2009; Van Beveren et al., 2024). Economically, mackerel caught within Canadian waters supported commercial fisheries for decades and served as a primary bait fish of high‐value species like bluefin tuna and American lobster (DFO, 2022). Moreover, mackerel hold cultural importance as a popular species for recreational fishing (Brushett et al., 2019) and as a traditional food for many Indigenous communities, such as Mi'kmaq, who have relied on Amalamaq (mackerel) for thousands of years (Hamelin et al., 2022). Despite this species' ecological, cultural and economic significance, West Atlantic mackerel biomass is in decline (DFO, 2025). The northern (Canadian) contingent has declined to such an extent that both the commercial and bait fisheries were closed in 2022, after earlier reductions in total allowable catch (DFO, 2025; Van Beveren, Boudreau, et al., 2023). The stock is currently characterized by a low‐spawning stock biomass, poor recruitment and collapsed age structure (DFO, 2025). Additionally, water temperatures have steadily increased across mackerel's habitat since the late 1990s (Bernier et al., 2018), potentially driving changes in mackerel distribution and stock dynamics (DFO, 2025; Overholtz et al., 2011; Brosset et al., 2020). To improve management and recovery strategies, it is crucial to gain an in‐depth understanding of movement ecology and habitat use of this mackerel stock, particularly in the face of a rapidly changing environment.

Expanding the use of biotelemetry to study mackerel can help fill critical knowledge gaps that could not be addressed by classic fishery‐dependent or fishery‐independent surveys, particularly related to migration dynamics, spawning site fidelity and spatial distribution (Crossin et al., 2017; DeCelles & Zemeckis, 2014). However, a few telemetry studies have been conducted for this species, largely due to the challenges associated with surgical tagging methods. Surgical tagging is an intrusive process (Cooke et al., 2011; Lower et al., 2005), with outcomes influenced by factors like handling practices, environmental conditions and individual fish characteristics such as size and condition (Jepsen et al., 2002). Mackerel are particularly vulnerable to tagging‐related injuries due to their sensitivity to handling and tendency to lose scales (Anders et al., 2021; Lockwood et al., 1983). Tagging procedures also often require an anaesthetic; however, its efficacy depends on both biological (e.g. species and size of fish) and abiotic factors (e.g. water temperature, pH and salinity) (Matsche, 2011; Treves‐Brown, 2000). For example, tricaine methanesulfonate (MS‐222) is effective for buccal‐pumping fish that actively pump water over their gills while anaesthetized but less suitable for ram ventilators, like tuna (Neiffer & Stamper, 2009) and mackerel (Roberts, 1975). Although buccal pumping has been observed in mackerel (Holeton et al., 1982; Boutilier et al., 1984), immersion in chemical anaesthesia may impair effective gas exchange as the fish become immobilized, increasing the risk of suffocation or respiratory alkalosis (Boutilier et al., 1984; Neiffer & Stamper, 2009). Chemical anaesthesia also has several disadvantages, including the long withdrawal times and negative effects on key behavioural and kinematic components involved in escape responses (Anderson et al., 1997; Kim et al., 2017; Ortuño et al., 2002). Residual chemical concentrations can be highly problematic for predation‐prone species like mackerel; even short‐term effects on responsiveness, directionality and swimming ability can increase the susceptibility to predation (Domenici, 2010).

Electroanaesthesia is a promising alternative for immobilizing mackerel during surgical tagging, offering rapid loss of movement control or response to stimuli without the withdrawal time and other negative side effects of chemical anaesthetics (Balazik et al., 2013; Durhack et al., 2020; Hudson et al., 2011; Kim et al., 2017; Reid et al., 2019; Soltani & Mirzargar, 2013). This technique works by applying a constant electrical current to induce immobilization, leading to a full loss of equilibrium and reactivity to external stimuli (Reid et al., 2019). Its efficacy has been demonstrated in several freshwater species, including the common carp (*Cyprinus carpio*; Monsef Rad et al., 2016; Kim et al., 2017), brown bullhead (*Ameiurus nebulosus*; Kim et al., 2017), lake trout (*Salvelinus namaycush*; Faust et al., 2017) and largemouth bass (*Micropterus nigricans*; Trushenski & Bowker, 2012; Kim et al., 2017). Although its application in marine species is still emerging, electroanaesthesia shows promise for improving fish welfare and refining surgical tagging techniques in mackerel.

The aim of this study was to test the use of electroanaesthesia as a novel method to immobilize Atlantic mackerel, with the broader goal of supporting the use of biotelemetry for studying this species. In a lab‐based study, we first observed the effectiveness of electroanaesthesia administered by electric fish handling gloves, qualitatively assessed wound healing to understand mackerel responses to ventral incisions and tag insertion, and evaluated potential factors [i.e. handling time (HT) and total length (TL)] influencing post‐tagging survival. Building on these results, we then applied the refined methods in the field, where wild mackerel were surgically tagged, released and tracked within an embayment to monitor survival in a natural environment. By addressing key methodological challenges and providing insight into post‐tagging survival rates for mackerel, this study provides an essential baseline for future biotelemetry studies on this species.

## 2 METHODS

### 2.1 Indoor pool tank experiment

#### 2.1.1 Animal care and pool tank conditions

Mackerel [fork length >26.8 cm (minimum retention length)] were sourced offshore Sambro, Nova Scotia, Canada, in October 2023 using standard hook‐and‐line fishing methods in compliance with a collection permit granted by Fisheries and Oceans Canada (DFO). Approximately 200 fish were transported by land and held in the Dalhousie Aquatron Pool Tank (Dalhousie University, Nova Scotia, Canada); a 684,050‐L circular tank (15.24 m diameter × 3.72 m depth) full of partially filtered seawater flowing at a rate of ~120 L/min pumped from the Northwest Arm, Nova Scotia, and filtered using sand and UV filters. Throughout the experiment, water temperature ranged from 11 to 16°C, varying with ambient seawater temperature. The tank was kept in a 12‐h light–dark photoperiod with 100% oxygen saturation. Fish were fed a commercial‐grade salmonid diet (3‐mm pellet size) thrice a day and given 3 weeks to acclimate to laboratory conditions before treatment. Any mortalities during this time were removed from the tank.

#### 2.1.2 Tagging equipment

Dummy tags were made of Delrin plastic (polyoxymethylene), cut into 28.5 × 7‐mm rods to simulate the size of a suitable acoustic transmitter for this size of fish. The edges were sanded down and marked with an identification (ID) number. Tagging equipment and tags were sterilized in 2% glutaraldehyde and rinsed in a veterinary‐safe 0.9% saline solution immediately before tagging.

#### 2.1.3 Tagging procedure

Mackerel that survived the acclimation period were fasted for 24 h before the tagging procedures began as recommended by the Canadian Council on Animal Care (Batt et al., 2005). The mackerel were corralled into a small area of the tank using a seine net for easy access with a dip‐net, and 41 individuals were netted for tagging. To facilitate the procedure, fish were temporarily transferred to a 100‐L holding tank filled with the same partially filtered seawater aerated with an air stone. To minimize crowding (e.g. Huse & Vold, 2010) and ensure easy access for the tagger, netters placed only five to seven fish in the holding tank at a time before replenishing it as tagging progressed. The first individual was netted from the holding tank and transferred to a foam‐topped tagging table using Electric Fish Handling Gloves (Smith‐Root, Inc., Vancouver, WA, USA). The anode glove (+ electrode) held the fish at its anterior end and the cathode glove (− electrode) at its posterior end, creating a closed electrical circuit, delivering a constant direct current (Reid et al., 2019). Following methods similar to those described in Reid et al. (2024), the current strength was gradually increased by individual increments until fish exhibited tetany, characterized by full‐body muscle contractions followed by relaxation, which was used as a proxy for complete immobilization (Reid et al., 2024; Summerfelt & Smith, 1990). Complete immobilization was achieved at 10 mA.

A recirculating pump delivered aerated saltwater through the fish's mouth and over its gills to ensure continuous irrigation. TL (cm) was measured to the nearest 0.5 cm, and dummy tag ID was recorded. A small (~10 mm) incision was made on the ventral midline, a tag was inserted into the intracoelomic cavity and the incision was closed using a single Ethilon 5–0 monofilament nylon suture. While still being immobilized with the electric gloves, the tagged fish was placed in a 100‐L recovery tank and HT (s) was recorded. HT was defined as the duration from when a fish was removed from the holding tank until it was placed in the recovery tank. The fish was monitored for abnormal behaviour (e.g. impaired swimming ability) or injury, then netted and returned to the pool tank. The electric gloves were kept at 10 mA, and the same tagging procedure was followed for the remaining 40 mackerel. Tank conditions were the same as the pre‐tagging conditions, and the fish were fasted for another 24 h to maintain water quality and promote energy allocation to recovery instead of digestion (Batt et al., 2005).

#### 2.1.4 Post‐tagging survival analyses

Fish were monitored across 40 days and observed daily for mortalities. Deceased individuals were removed from the tank the same day, and external signs (i.e. skin infections) that could help explain the cause of death were noted. At the end of the 40‐day monitoring period, total mortalities were quantified, and the remaining fish were killed with a lethal dose of MS‐222 (>250 mg/L). To assess one methodological and one biological factor influencing survival after surgical tagging, we analysed time to mortality or study endpoint (40 days post‐tagging) as a function of HT and fish size (TL). For each factor (HT and TL), fish were grouped as either above or below the median HT (103 s) and the median TL (32 cm). A survival analysis was conducted for both factors using a Cox proportional hazard test (R package ‘survival’; Therneau & Grambsch, 2000; Therneau, 2024) to evaluate their effects on survival time in days (Goel et al., 2010). Kaplan–Meier survival curves were generated (R package ‘survminer’; Kassambara et al., 2021) to illustrate survival probability over time among tagged individuals.

To further investigate the predictors of mortality, we performed a multiple logistic regression (1 = survived, 0 = mortality event), modelling survival probability as a function of HT and TL. This approach assessed whether handling duration and fish size influenced the likelihood of mortality independent of time‐based survival analysis. The model was fitted using a generalized linear model with a binomial error distribution and logit link function:

$$\mathrm{Log}\left(\frac{p\left(m=1\right)\;}{\;1-p\left(m=1\right)}\right)=\beta 0+\beta 1\cdot \mathrm{TL}+\beta 2\cdot \mathrm{HT}$$

where $p\left(m=1\right)$ represents the probability of survival and β_1_ and β_2_ are the estimated coefficients for TL and HT, respectively. Although we use a significance threshold of *p* <0.05, it is recognized that the *p*‐value alone does not quantify effect size or result importance, and therefore, we considered the broader context, including relevant literature, when evaluating results (Wasserstein & Lazar, 2016). Survival probabilities were predicted using the logistic regression model to illustrate the relationship with HT and TL.

#### 2.1.5 Wound healing analysis

**FIGURE 1:**
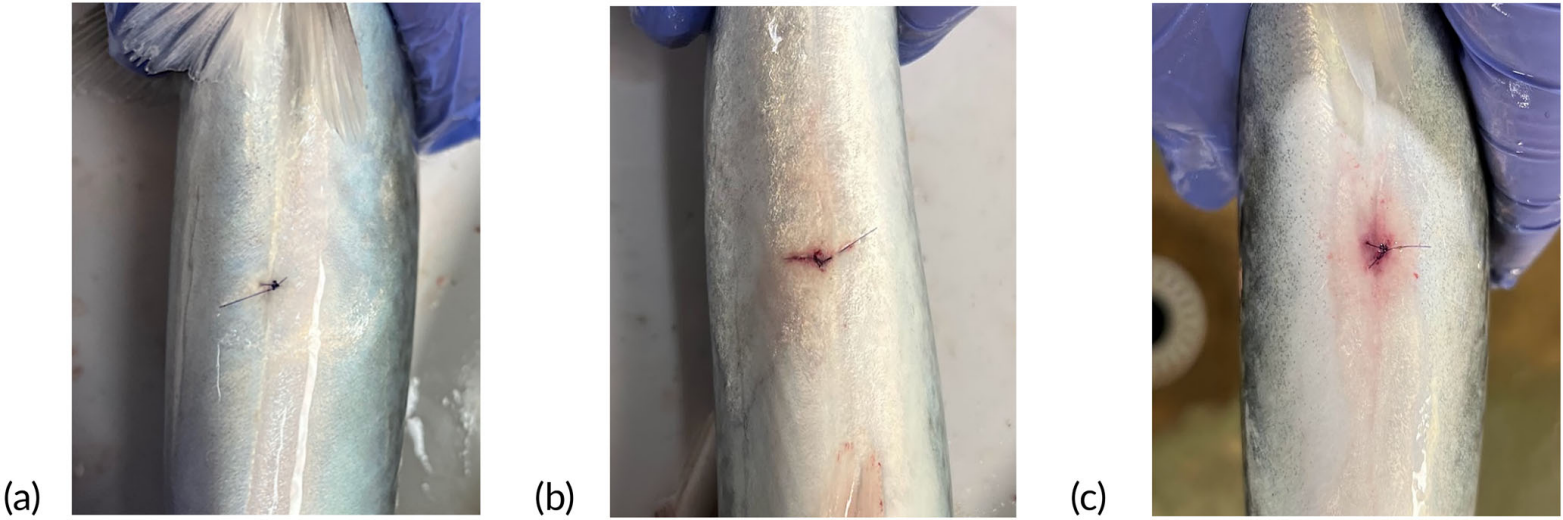
Sutured closed incision sites on the ventral side of Atlantic mackerel (*Scomber scombrus*) post‐surgical tagging procedure: (a) fully healed, (b) healed with minor redness and (c) severe redness.

Images of the incision sites were obtained immediately after fish were removed from the tank for visual assessment of healing after surgical tag insertion. Wound condition was grouped into one of the following categories based on physical observation: (1) fully healed, (2) healed with minor redness or (3) severe redness (Figure 1). Dummy tags were removed using a scalpel to identify the individual. The proportion of mackerel by wound (1–3) and survival (survived or deceased) category was calculated across all 41 tagged fish. See Table  for the metadata associated with lab‐tagged fish.

### 2.2 Field experiment

#### 2.2.1 Study site

**FIGURE 2:**
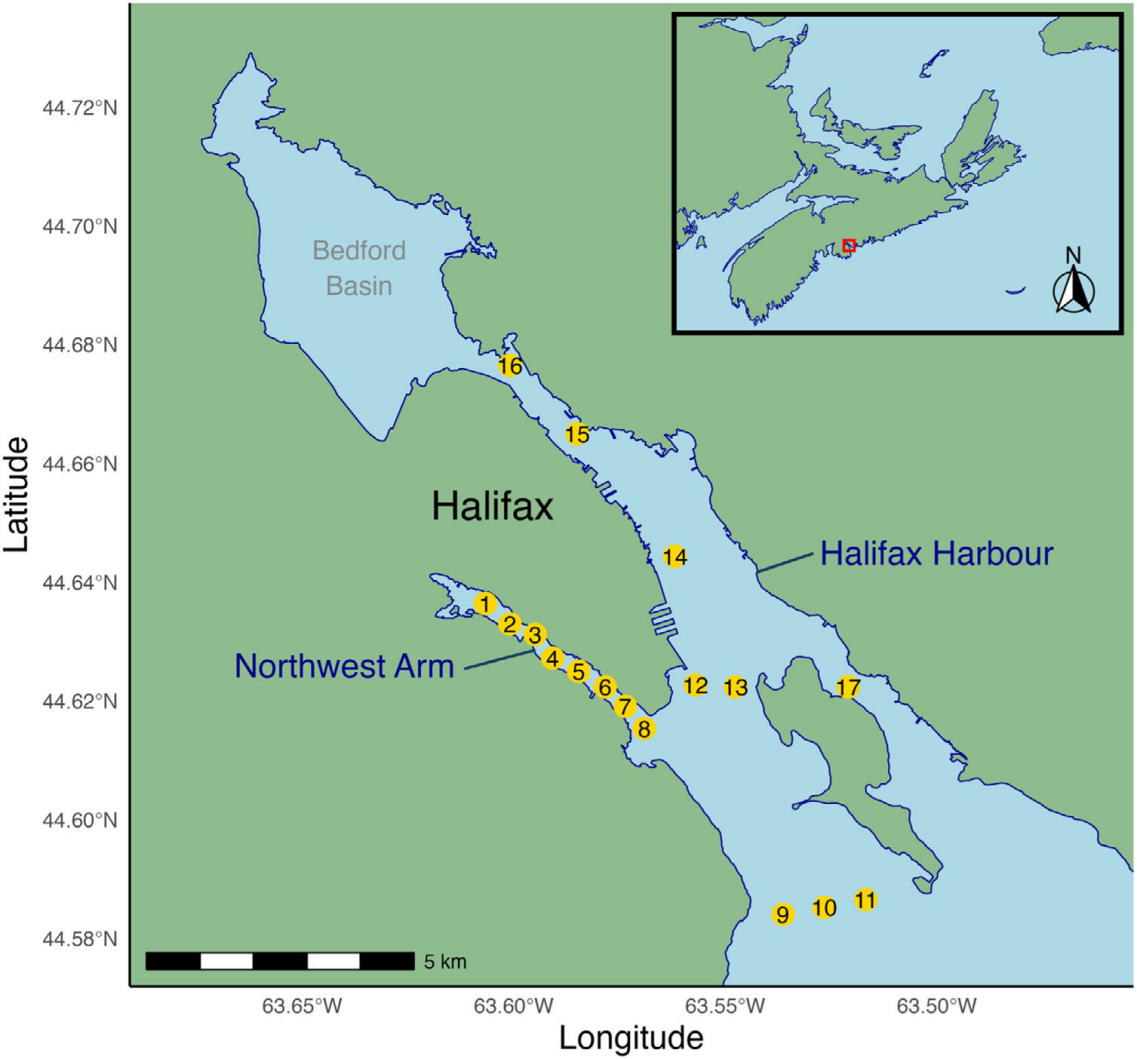
Map of the study area in Halifax, Nova Scotia, highlighting the Northwest Arm and Halifax Harbour. Yellow circles indicate the locations of 17 stationary acoustic receivers (VR2, Innovasea, Bedford, Nova Scotia, Canada), each labelled with its corresponding station number. The inset map shows Halifax (red square) on the east coast of Canada.

The field‐based study was conducted along the southern shore of Nova Scotia within the Northwest Arm and Halifax Harbour, a coastal system connected to the Atlantic Ocean (Figure 2). In May 2024, 17 stationary acoustic receivers (VR2, Innovasea, Bedford, Nova Scotia, Canada) were deployed throughout the study site. Eight receivers were positioned linearly within the Northwest Arm to detect the movement of mackerel as they migrated from the inner release site (receiver 1) towards the mouth of the inlet (Kraus et al., 2018). The remaining nine receivers were distributed throughout the Halifax Harbour to monitor survival across a broader area beyond the Northwest Arm.

#### 2.2.2 Tagging procedure with methodological refinements

Methodological adjustments were implemented when working in the field, informed by empirical observations and logistical insights from the lab‐based tagging procedures, with the goal of improving mackerel welfare and post‐release survival. In July 2024, mackerel were captured via angling in the Northwest Arm using Sabiki rigs with five barbless hooks per rod. Angling was chosen over alternative capture methods (e.g. weirs, trawls or nets) to maximize the welfare of the mackerel before tagging by minimizing scale damage. The fish were dehooked into floating lobster crates and then transferred to an ~450‐L circular tank (instead of a rectangular tank to allow schooling) on shore filled with aerated seawater, which served as the holding tank for tagging. A total of 50 mackerel were tagged following the same general methods used in the first half of the study, with some refinements: no nets were used for any capture or transport, and instead of being transferred to the tagging table while immobilized using the electric gloves, each mackerel was caught by hand by a researcher wearing vinyl gloves and placed on a foam‐topped tagging table for surgery. Once on the table, the fish was then anaesthetized with the electric fish handling gloves and tagged with HP9 transmitters (9 mm diameter, 39 mm length, 6.8 g in air, Thelma Biotel, Trondheim, Norway). See Table S2 for metadata associated with field‐tagged fish. Due to the larger tag size (relative to dummy tags in the laboratory trial), incisions were closed using two interrupted sutures. Once the surgery was completed, contact with the electric gloves ceased, and the fish was returned to a recovery tank by the researcher wearing the vinyl gloves. The tagged mackerel were monitored for 1 h before being released back into the Northwest Arm upstream of receiver 1 (Figure 2).

#### 2.2.3 Post‐tagging survival analyses

Detection data were downloaded from receivers through 11 November 2024 and processed to remove false detections and infer the fate of tagged animals. False detections were filtered using temporal patterns at each receiver station using the ‘glatos’ package in R (Holbrook et al., 2021). Following the protocol outlined by Pincock (2012), a detection was identified as a false positive if it occurred only once at a receiver within a 1‐h period and was not preceded by any detections from the same individual in the prior 60 min. A total of 400 detections (0.72%) were filtered from the dataset using this rule. Tagging‐related mortality was inferred from this dataset based on the knowledge that mackerel are highly migratory species, with immediate cessation of movement or prolonged stationarity considered indicative of death. Two criteria were used: (1) absence of detections post‐release, consistent with the flow chart developed by Villegas‐Ríos et al. (2020), and (2) continuous detections at a single receiver station for an extended period, following the approach of Melnychuk et al. (2013) and Klinard and Matley (2020). An operational threshold of five consecutive detections at a single receiver within 24 h, followed by no further detections, was used to classify mortality events and identify mortality dates. Detection data from Ocean Tracking Network receivers outside the study area were used to validate survival classifications, confirming mortality when no further detections occurred and supporting survival of individuals if detected beyond the study site. Those not detected outside the study site, but also not meeting mortality criteria, were considered right censored, meaning their last known detection indicated they were alive but their fate beyond that point was unknown (Berwick et al., 2004).

Kaplan–Meier survival analysis was used to estimate survival probabilities over time, incorporating right‐censored data to account for uncertainty in final fate. Three fate categories were defined: confirmed mortality (event = 1), confirmed survival (event = 0) and unknown (right censored) (Berwick et al., 2004; Goel et al., 2010). Kaplan–Meier survival curves were constructed to illustrate the survival probabilities of mackerel in the laboratory study compared to the field study using the refined methods, over time. A log‐rank test was used to evaluate if survival differed between the two groups (Goel et al., 2010).

R, version 4.4.2, was used for all statistical analyses in this study (source code available at https://github.com/CaliyenaBrown/electric-mackerel2.0).

### 2.3 Ethics statement

This work was conducted in accordance with guidelines established by the Canadian Council on Animal Care. All capture and tagging were conducted in compliance with Animal Care Permits 1039086, 1039086 and 1040763 and a Section 52 Permit 371740.

## 3 RESULTS

### 3.1 Indoor pool tank experiment

#### 3.1.1 Post‐tagging survival analyses

**FIGURE 3:**
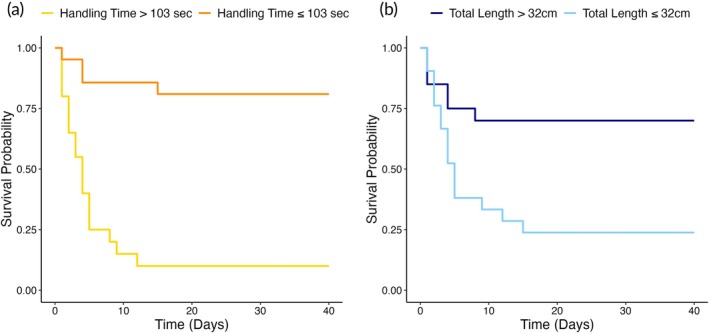
Kaplan–Meier survival curves of the effect of (a) handling time [HT (s)] and (b) total length [TL (cm)] on the survival probability of surgically tagged Atlantic mackerel (*Scomber scombrus*) over 40 days. (a) Fish were grouped based on the median HT (103 s), with individuals handled for ≤103 s (orange) exhibiting significantly greater survival probability compared to those handled for >103 s [yellow; Cox proportional hazards test: HR = 0.100, 95% CI (confidence interval): 0.03–0.30, *p* < 0.001]. (b) Fish were grouped based on the median TL (32 cm), with individuals >32 cm (dark blue) exhibiting significantly greater survival probability than those ≤32 cm (light blue; HR = 3.374, 95% CI: 1.31–8.69, *p* = 0.012).

A total of 22 of the 41 tagged fish (~53.6%) died within the first 2 weeks after surgery, all of which had skin lesions around the head and caudal peduncle, and signs of infection at the time of recovery. Cox proportional hazards tests revealed that HT significantly affected mackerel survival [HR = 0.100, 95% confidence interval (CI): 0.03–0.30, *p* < 0.001], indicating that fish handled for ≤103 s had a reduced risk of mortality compared to those handled for longer durations (Table S3). Fish handled for over 103 s exhibited a sharp decline in survival, with about 95% of mortality occurring within the first 10 days. By contrast, fish handled for ≤103 s had a higher survival probability, which stabilized at about 75% after 2 weeks (Figure 3a). Similarly, TL had a significant effect on survival (HR = 3.374, 95% CI: 1.31–8.69, *p* = 0.012), suggesting that smaller mackerel (≤32 cm) faced a higher risk of mortality compared to larger individuals (Table S4). Mackerel measuring ≤32 cm experienced greater mortality, with survival probability decreasing to less than 25% within the first 2 weeks. In contrast, larger individuals (>32 cm) exhibited greater survival, with probabilities stabilizing just below 75% across the monitoring period (Figure 3b). The survival curves demonstrated that under optimal conditions (low HT, high TL) survival stabilized within about a week, whereas under suboptimal conditions, mortality continued into the second week (Figure 3).

**FIGURE 4:**
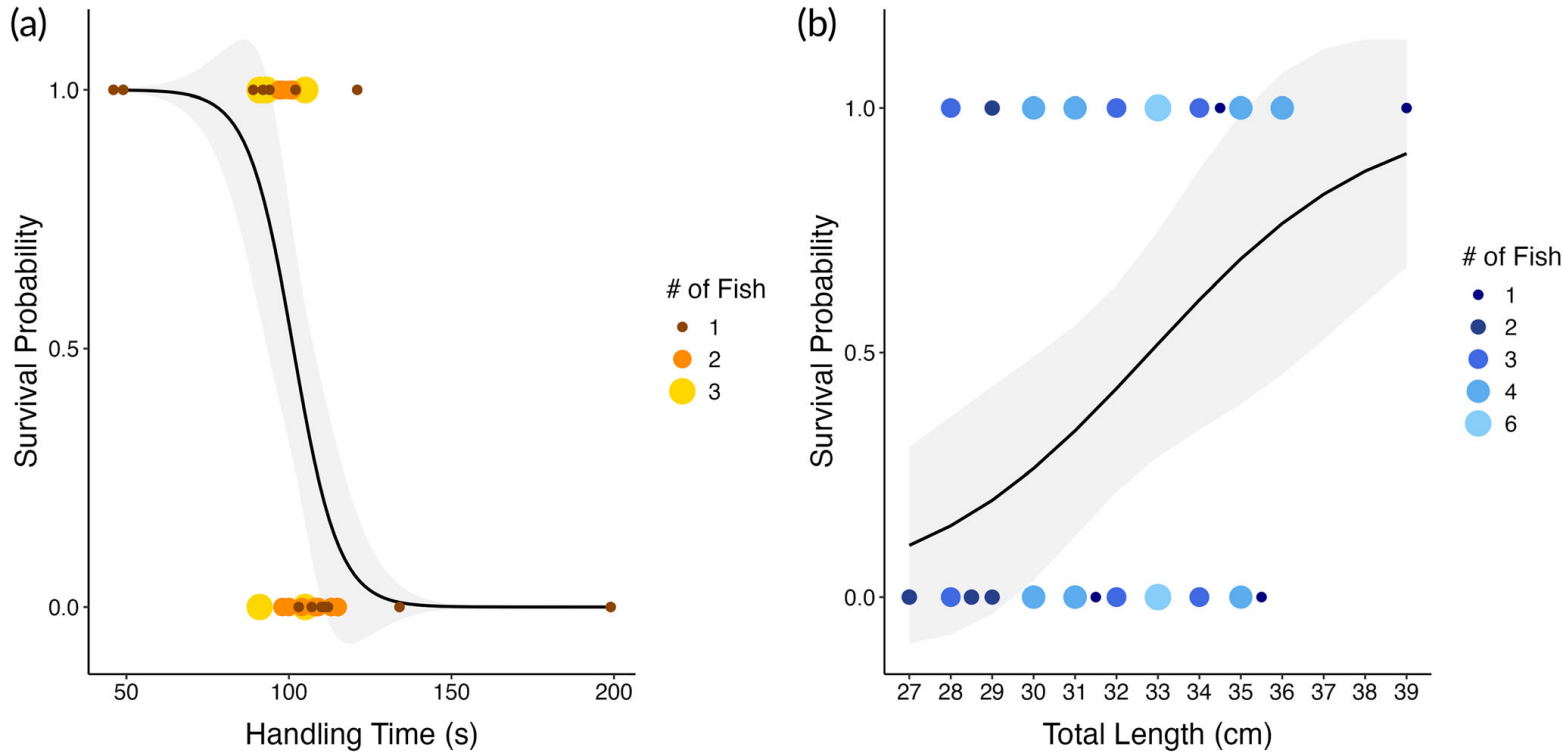
Logistic regression curves illustrating the effect of (a) handling time [(HT s)] and (b) total length [TL (cm)] on the survival probability of surgically tagged Atlantic mackerel (*Scomber scombrus*). Model‐predicted survival probabilities are shown as a function of HT and total length, with grey ribbons indicating 95% confidence intervals. Each point represents an individual fish, with point size and colour scaled to the number of fish at each value. (a) Survival probability declined significantly with increasing HT [odds ratio = 0.87, 95% CI (confidence interval): 0.75–0.95; *p* = 0.0192], whereas TL (total length) had a marginally non‐significant positive effect (odds ratio = 1.44, 95% CI: 1.00–2.21; *p* = 0.0577).

Multiple logistic regression (Figure 4) revealed a significant effect of HT on survival probability (*z* = −2.341, *p* = 0.0192), with an odds ratio of 0.87 (95% CI: 0.75–0.95; Table S5), indicating that longer handling durations reduced the likelihood of survival in mackerel. Conversely, TL had a marginally non‐significant effect on survival (*z* = 1.898, *p* = 0.0577). However, the estimated odds ratio of 1.44 (95% CI: 1.00–2.21; Table S5) suggests that larger mackerel had about 44% higher odds of surviving for each additional centimetre in length, supporting the observed trend from the size class–specific survival analysis. The logistic regression curves (Figure 4) illustrate these relationships; survival probabilities exceeding 50% were generally associated with HTs <~100 s (Figure 4a) and total lengths >~33 cm (Figure 4b), whereas probabilities above 75% were limited to individuals combining short HTs and larger body sizes. Among survivors, the median TL was 34 cm (95% CI: 33–35 cm) and the median HT was 94 s (95% CI: 92–100 s), reinforcing that post‐tagging survival was more likely for larger fish and those handled quickly. Particularly, some smaller individuals (<30 cm) did survive, but typically when HTs were short (e.g. tag ID: 38, TL = 28 cm, HT = 97 s, and tag ID: 46, TL = 29 cm, HT = 49 s; Table S1), highlighting the importance of minimizing handling duration across all size classes.

#### 3.1.2 Wound healing analysis

Qualitative assessment of the wound sites revealed that 43.9% of tagged mackerel exhibited fully healed incisions, 48.8% exhibited minor redness and 7.3% exhibited severe redness, indicating that most individuals healed completely or exhibited only minor signs of irritation after surgical tag implantation. Among the fish that survived the 40‐day monitoring period, 57.9% had fully healed incisions and the remaining 42.1% exhibited minor redness. None of the survivors exhibited severe redness at the end of the study. Among the mortalities, 31.8% had fully healed wounds, 54.5% exhibited minor redness and 13.6% exhibited severe redness. All individuals with severe redness died during the monitoring period and had HTs above the median (103 s). Although their total lengths were more variable, ranging from 28.5 to 34.5 cm, these fish were smaller on average (Figure S1). We found no evidence of tag loss.

### 3.2 Field experiment

**FIGURE 5:**
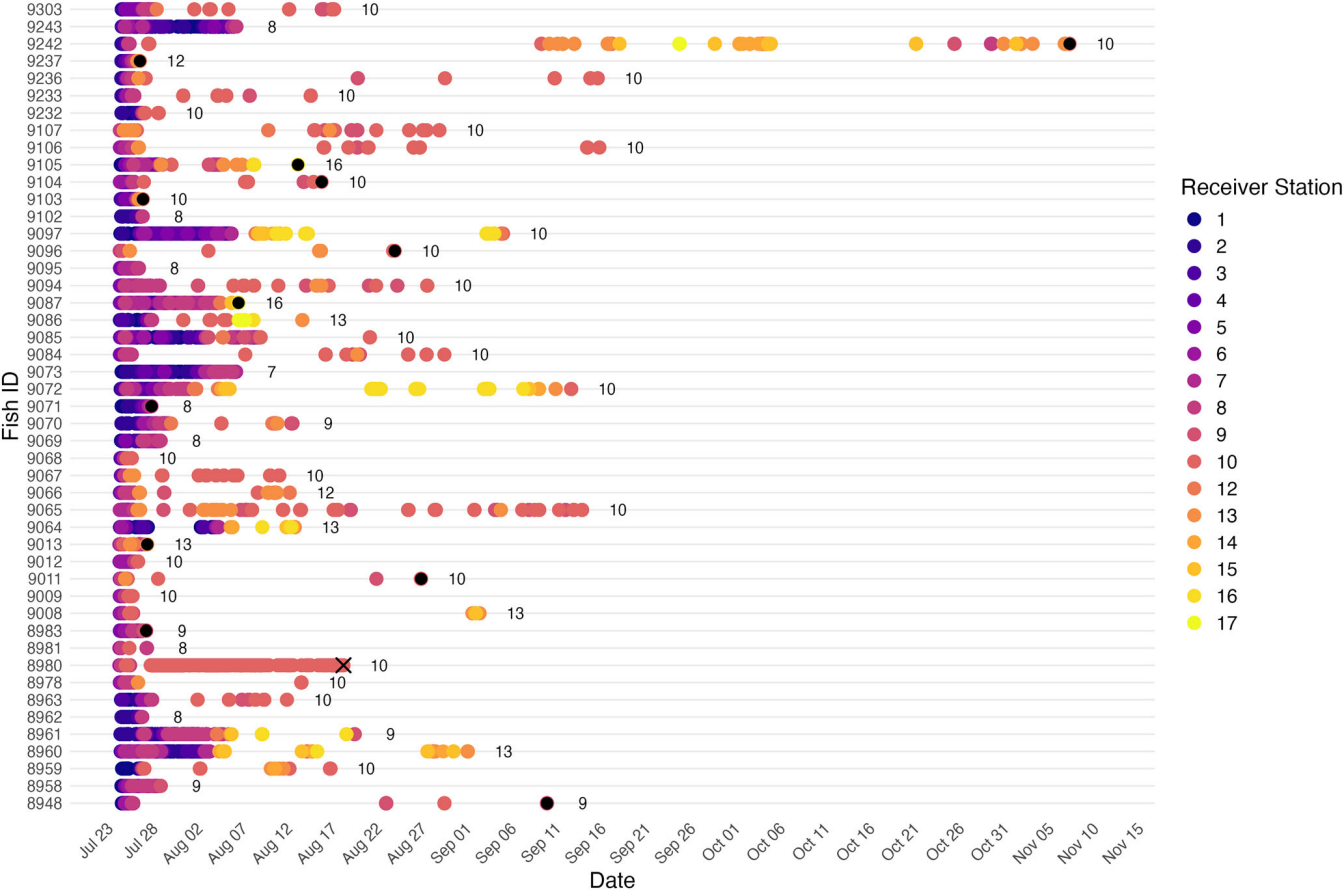
Abacus plot showing individual detections of tagged Atlantic mackerel (*Scomber scombrus*; *n* = 47 of the 50 tagged fish), exhibiting movements from the inner Northwest Arm (receiver 1) towards the mouth of the Northwest Arm (receivers 8) and out into the Halifax Harbour (receivers 9–17) over time. Each point represents a detection and colour corresponds to receiver station. Numbers to the right of the final detection point indicate the receiver station of the final detection of that individual within the study area. Black‐filled detection points mark the final detection of right‐censored individuals (fate unknown, *n* = 12), who were not confirmed dead or detected outside the study area. ID 8980, with the black X over the final detection point, identifies the potential tagging‐related mortality based on subsequent detections at the same receiver station over 24 h, indicating no horizontal movement.

Of the 50 tagged mackerel, four individuals (8%) were classified as mortalities during the field study. Three fish were never detected post‐release and were therefore presumed to have experienced tagging‐related mortality immediately after release based on criterion 1. One fish (ID 8980) met criterion 2 and exhibited continuous detections at receiver station 10 across 24 days, followed by a complete cessation of detections, also indicative of post‐tagging mortality (Figure 5). This individual was not detected on any acoustic receivers outside of the study site.

**FIGURE 6:**
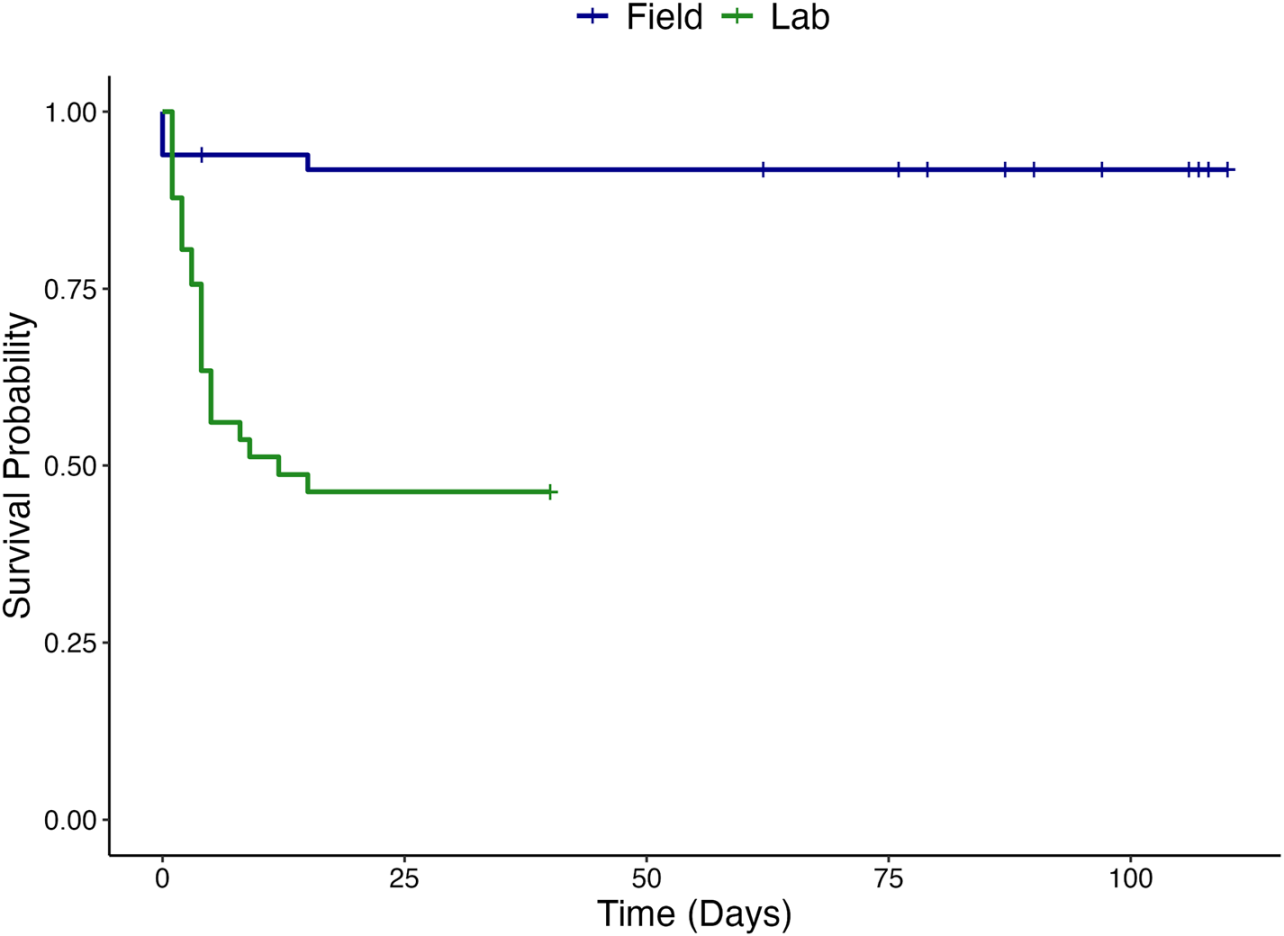
Kaplan–Meier survival curves illustrating the survival probability of surgically tagged Atlantic mackerel (*Scomber scombrus*) in the field (*n* = 50) and laboratory studies (*n* = 41). The in‐lab survival curve ends at 40 days, reflecting the duration of the laboratory study period. Lab‐tagged fish exhibited a sharp decline in survival within the first 2 weeks, whereas field‐tagged fish exhibited a high survival probability (~92%) across the 110‐day monitoring period. The survival difference between groups was significant (log‐rank test: χ^2^ = 21.7, *df* = 1, *p* < 0.001). Right‐censored individuals in the field (*n* = 12) are indicated by vertical ticks along the survival curve.

The log‐rank test showed a highly significant difference in survival probability between the lab‐ and field‐tagged mackerel (χ^2^ = 21.9, *df* = 1, *p* < 0.001), with lab‐tagged mackerel exhibiting substantially greater post‐tagging mortality than the field mackerel. Of the 41 fish tagged in the laboratory, 22 (53.7%) died, with most mortalities occurring within the first 2 weeks. In contrast, only 4 of the 50 field‐tagged fish (8%) died over the study period. Survival probability declined most sharply within the first few days post‐release for both lab‐ and field‐tagged mackerel. However, lab‐tagged fish continued to experience mortality into the second week, with survival stabilizing at about 46%, whereas field‐tagged mackerel had only one additional mortality after the first week and maintained a stable survival probability of about 92% for the remainder of the study (Figure 6).

## 4 DISCUSSION

Biotelemetry is a powerful tool for understanding population dynamics and migration patterns of exploited species, playing a valuable role in fisheries management (Crossin et al., 2017). For example, annual passive integrated transponder tagging experiments on Northeast Atlantic mackerel have provided insights into size‐dependent migratory behaviour between spawning and feeding areas (Ono et al., 2022). Such research would be instrumental in enhancing our understanding of the mackerel stocks in the Northwest Atlantic, particularly given the recent drastic reductions in the commercial and bait fisheries, which have reduced fishery‐dependent data sources. Moreover, mackerel stock assessments rely on the assumption that annual egg surveys accurately reflect spawning stock biomass; however, some mackerel may spawn across a much broader range than is surveyed, and this spatial plasticity in spawning migration is still poorly understood (Van Beveren, Plourde, et al., 2023). Incorporating biotelemetry into mackerel research has the potential to fill critical knowledge gaps on its movement ecology, driven by the absence of expensive large‐scale scientific surveys targeting the adults, the poor quality of fishery statistics and limitations in egg survey coverage. However, to generate reliable data from biotelemetry, species‐specific tagging methods are needed to maintain animal welfare and minimize post‐release mortality (Bridger & Booth, 2003; Cooke et al., 2011). To develop these methods for mackerel, we first conducted an in‐lab pilot study and then applied refined methods in an at‐sea tagging study to assess immediate post‐tagging survival and establish best practices for tagging this species.

Over half of the lab‐tagged mackerel died within 2 weeks post‐surgery, and most of these mortalities were associated with visible skin lesions, independent of the tagging wound, and signs of infection. The survival analyses revealed that longer HTs significantly reduced the probability of mackerel survival in captivity. This finding is likely due to the vulnerability of scombrids to handling, because they lack the protection of large scales and rely on their protective mucous layer, making them particularly susceptible to scale loss (Bar et al., 2015; Holeton et al., 1982; Lockwood et al., 1983). Therefore, handling with the electric gloves, which had a knit exterior, may have contributed to skin damage and increased susceptibility to infection in the tank, leading to mortality. Although stress indicators and cause‐of‐death assessments were not conducted, this interpretation is supported by previous work by Hamre (1970), who identified that the extent of handling was the principal cause of mortality in surgically tagged mackerel. Mucous loss has also been linked to a higher risk of infection (Svendsen & Bøgwald, 1997), which has been associated with high mortality rates in other fish species (Anders et al., 2021; Lockwood et al., 1983). Additionally, many of the mackerel brought into the laboratory for the pilot study were in visibly poor condition upon arrival, and substantial mortality (~130 of ~200 individuals) occurred during the acclimation period before the experiment. Although fish were given time to acclimate and begin feeding before treatment began, it is likely that some infections and skin damage observed post‐tagging were linked to pre‐existing injuries or stress from the initial capture and transport process rather than the tagging procedure alone.

Beyond the effects of handling, variation in mackerel size emerged as a key factor influencing post‐tagging survival. Although logistic regression revealed a marginally non‐significant effect (*p* = 0.0577), the overall trend and survival analysis indicated that shorter mackerel (≤32 cm) experienced substantially greater and earlier mortality, with most deaths occurring within the first 2 weeks, whereas larger mackerel had greater survival probabilities across the 40‐day monitoring period. These findings contrast with Bar et al. (2015), who reported that smaller scombrids (<1 kg) survived capture and transport, whereas the larger individuals (TL > 60 cm) did not. Similarly, larger wild‐caught rainbow trout had elevated markers of acute stress (i.e. plasma cortisol and lactate levels) after prolonged landing and handling than did smaller counterparts (Meka & McCormick, 2005). Together, these studies suggest that vulnerability to mortality may be size‐specific and stage‐dependent, with larger fish being more susceptible to capture‐related stress and smaller fish more vulnerable to post‐tagging complications. In particular, smaller mackerel may face a heightened risk of internal trauma due to their limited body cavity space, increasing the likelihood of tag‐induced organ damage or impaired recovery (Brown et al., 2011), especially when all fish receive the same‐sized tags. Future studies should investigate postmortem internal injury or tag placement effects to determine the mechanisms underlying early mortality in smaller individuals. Additionally, to better understand the drivers of mortality, future studies should include control groups, such as an only handled group, and a handled and anaesthetized group, to isolate the effect of the surgical tagging.

The qualitative assessment of incision sites revealed promising outcomes for wound healing in mackerel after surgical tag implantation. Among surviving individuals, most exhibited either fully healed incisions or only minor redness, suggesting that mackerel possess a robust capacity for wound healing despite their delicate scales. In contrast, a small proportion of tagged fish exhibited severe redness at the incision site, and particularly, all of these individuals died before the end of the monitoring period. Stress‐induced immunosuppression is a well‐documented phenomenon in fish, where both chronic stress from captivity and acute injury–related stress can reduce immune function and impair healing (Huntingford et al., 2006; Noble et al., 2012). In mackerel, such stressors may have been further exacerbated by confinement in tanks, which has been shown to increase disease susceptibility in other scombrid species (Bar et al., 2015). Although healing appeared to progress slowly during the initial post‐tagging period, most incisions nearly or fully healed by day 40, which was consistent with Bar et al. (2015), who reported evidence of skin recovery in scombrid species within 2 weeks of antibiotic treatment in captivity.

Electroanaesthesia using electric fish handling gloves appeared effective for sedating mackerel during surgical tagging, as indicated by consistent immobilization and rapid behavioural recovery observed in the pilot study. Upon glove removal, the mackerel immediately regained full mobility and resumed schooling after release into the pool tank. The lack of visible muscular impairment and immediate onset and termination of sedation suggest that electroanaesthesia minimizes recovery time and preserves key behavioural and kinematic functions (i.e. predation escape) in mackerel. This near‐instantaneous recovery contrasts with chemical anaesthesia, where fish recovery can take 1 to >60 min (Topic Popovic, 2012). Although these observations were qualitative, the gloves provided an efficient and repeatable means of immobilization, aligning with previous work on other species showing that electroanaesthesia can be a safe and reversible form of sedation (Reid et al., 2019; Trushenski & Bowker, 2012). Nonetheless, an important limitation is the absence of physiological validation. Although behavioural responses were encouraging, including immediate resumption of normal swimming and schooling, the internal effects of electroanaesthesia remain unknown. Future studies should include physiological measures such as stress responses (i.e. blood chemistry and hormone levels) and directly compare electroanaesthesia and chemical anaesthesia to determine which method best supports mackerel welfare. Importantly, electroanaesthesia did not produce observable negative effects on immediate behaviour or survival, incision healing was successful in most cases and no tag expulsions were observed, indicating reliable tag retention. Despite concerns with the electric glove material, we argue that electroanaesthesia remains a promising alternative to chemical anaesthesia due to its rapid recovery time, suitability for ram ventilators and lack of negative side effects from chemicals (Durhack et al., 2020; Reid et al., 2019). These findings provided sufficient justification for the use of the electric gloves and surgical tagging methods in the field study.

The significant increase in post‐tagging survival observed in the field (92%) compared to the lab‐tagged mackerel (46%) highlights the effectiveness of the methodological refinements adopted following observations and insights from the pilot study. Additionally, survival is more likely in the natural environment, where fish are not subject to captivity‐related stressors, which has been associated with high mortality in other species (Marçalo et al., 2008). In particular, the field environment offers lower pathogen loads, more stable water quality and greater opportunity for natural recovery behaviours (e.g. active swimming, schooling), all of which likely contributed to the observed survival outcomes (Marçalo et al., 2008; Yanong, 2003). Survival probability declined most sharply within the first few days post‐release for both lab‐ and field‐tagged mackerel, suggesting immediate post‐tagging stress and surgical complications as the primary cause. However, only lab‐tagged fish continued to experience mortality into the second week, suggesting that prolonged mortality in captivity may also reflect chronic stress and secondary infections associated with confinement. This further supports the interpretation that both refined tagging methods and the absence of captivity‐related stressors contributed to improved survival in the field.

However, the interpretation of survival outcomes in the field includes limitations. Although our mortality classification criteria were based on established protocols for inferring mortality from acoustic detections (Klinard and Matley 2020), alternative explanations remain possible. For instance, fish that were never detected post‐release may have evaded detection due to tag failure (Bird et al., 2017). Predation is another potential source of uncertainty, and although we examined individual movements for patterns that could indicate predation events (e.g. rapid movements followed by prolonged stationary signals post‐excretion), confirmation was not possible without specialized predation‐detection tags. More definitive confirmation could be obtained using pressure (depth) sensors, predation and/or temperature sensors (Lennox et al., 2023). For example, depth profiles would help distinguish between a live fish that is remaining still and a dead fish resting on the seafloor, which typically produces flat, unchanging depth traces over time (Villegas‐Ríos et al., 2020). Incorporating such sensors in future studies would therefore strengthen the inference of mortality events and clarify ambiguous cases. Additionally, the 12 (24%) individuals that were censored in the survival analysis may have died outside of the receiver range, potentially leading to underestimated mortality. Nonetheless, the broad movement patterns observed in most fish and the lack of mortality signals outside of those identified suggest that the majority of tagged mackerel recovered successfully after surgery.

## 5 SUMMARY AND RECOMMENDATIONS

We developed novel tagging procedures for Atlantic mackerel in both laboratory and field settings. In the laboratory, survival was low, with higher mortality associated with longer HTs and shorter body lengths. However, most mackerel exhibited only minor redness or fully healed incisions by the end of the monitoring period. In contrast, field‐tagged mackerel experienced very few mortalities. Taken together, we provide recommendations for surgical tagging and electroanaesthesia techniques for Atlantic mackerel (Box 1). First, we recommend choosing capture methods that reduce scale damage, specifically angling instead of netting (Brownscombe et al., 2019), using barbless hooks (Cooke et al., 2022) and unhooking with minimal direct handling. In this study, dehooking was completed without direct contact, and instead, the fish were released from the hook into the holding tank with a quick twist and drop motion of the hook shank, eliminating manual handling. Additionally, no nets were used at any point to minimize scale loss and skin damage. It is important to release visibly unhealthy individuals or mackerel damaged during capture untagged (Brownscombe et al., 2019). This aligns with previous work showing that compromised individuals are more likely to die post‐tagging due to the cumulative stress of injury and the tagging procedure (Cook et al., 2019). We recommend tagging only larger individuals, despite the scientific value of tagging a wider range of sizes. Although some studies suggest larger fish may suffer higher mortality (Bar et al., 2015), this has been linked to stress during capture and transport, not the tagging itself. Our findings indicate that larger mackerel have higher survival post‐tagging. However, it is important to consider that the size threshold will also depend on tag size. Although electroanaesthesia at 10 mA was effective in this study, optimal voltage will vary with species, fish size and environmental conditions (Reid et al., 2019). Therefore, we recommend determining the appropriate voltage for effective immobilization by gradually increasing the current until tetany is achieved. Ensuring proper immobilization is critical for mackerel welfare and to minimize stress during handling (Reid et al., 2019). To limit skin damage from the electric fish handling gloves, we minimized contact with the electric gloves to only what is necessary to induce adequate sedation. Handling (i.e. movement between tanks, movement onto tagging tables) was instead done using smooth‐surfaced polyvinyl chloride or nitrile gloves, which have been shown to minimize skin damage in scombrids (Bar et al., 2015). Dividing these tasks, where one person administers electroanaesthesia and another handles the fish, maintains effective immobilization while reducing direct contact with abrasive gloves. Finally, cumulative HT should be minimized, ideally to less than a minute between capture and release. Extended air exposure increases stress and the likelihood of more severe injury (Cook et al., 2015). Across the laboratory and field components of our work, the learnings and refinements to methods contribute to a best‐practice framework for tagging Atlantic mackerel that maximizes fish welfare and improves the quality and reliability of biotelemetry data.

BOX 1 **Recommendations of best acoustic tagging practices for Atlantic mackerel, using electroanaesthesia with electric fish handling gloves.**

**Best tagging practices for Atlantic mackerel:**

- Opt for capture methods that minimize scale damage (i.e. angling over netting).
- Use barbless hooks to minimize mouth tearing.
- Minimize direct handling when dehooking.
- Release small (TL <29 cm) fish and individuals damaged during capture untagged.
- Use circular holding tanks to allow schooling and to reduce the risk of head collisions with tank walls.
- Do not use nets for capture from or transport between holding tanks; use smooth rubber gloves instead.
- Determine the appropriate voltage for effective immobilization.
- Minimize contact with electric gloves.
- Prioritize reducing cumulative HT.

## AUTHOR CONTRIBUTIONS

Caliyena R. Brown: project conceptualization; data collection; manuscript writing, preparation and editing; statistical analyses. Morgan L. Piczak: project conceptualization, funding acquisition, data collection, statistical analyses and manuscript editing. Elisabeth Van Beveren: provision of resources, manuscript validation and editing. John Batt: animal care, provision of resources, project management. Robert J. Lennox: supervision, project conceptualization, funding acquisition, statistical analyses and manuscript editing.

## FUNDING INFORMATION

Caliyena R. Brown and Robert J. Lennox were supported by Fisheries and Oceans Canada to conduct the laboratory study. Morgan L. Piczak is supported by the Liber Ero Fellowship Program, NSERC and Fisheries and Oceans Canada.

## Supporting information


**Supplementary Figure 1.**Relationships between wound condition and biological or handling metrics among surgically tagged Atlantic Mackerel (Scomber scombrus) that died within the 40‐day monitoring period. Boxplots show the distribution (from left to right) of days until death, handling time (sec), and total length (cm) for mackerel categorized by wound condition (a = fully healed, b = minor redness, c = severe redness) at time of recovery from laboratory tank. Only fish that died during the 40‐day post‐tagging monitoring period are included (*n* = 22). These exploratory comparisons were not tested statistically and were intended to visually assess trends.
**Supplementary Table 1.**Metadata of lab‐tagged Atlantic mackerel (Scomber scombrus) including tag ID fate, date of death, handling time (seconds), total length (cm), and wound condition category (a = fully healed, b = minor redness, c = severe redness). The shaded boxes demonstrate the end of the 40‐day monitoring period.
**Supplementary Table 2.**Metadata of field‐tagged Atlantic mackerel (Scomber scombrus) including fate: survived, censored, or mortality (not detected or deceased), date of death, date of last detection, and the receiver station of the last detection.
**Supplementary Table 3.**Results of the Cox proportional hazards model assessing the effect of HT (sec) on survival time of lab‐tagged Atlantic mackerel (Scomber scombrus). The analysis includes the log‐rank test statistic, hazard ratio (HR), 95% confidence interval (CI), and p‐value. The significant HR less than 1 indicates a decreased risk of mortality for fish handled for ≤ 103 seconds compared to those handled for longer durations.
**Supplementary Table 4.**Results of the Cox proportional hazards model assessing the effect of TL (cm) on survival time of lab‐tagged Atlantic mackerel (Scomber scombrus). The analysis includes the log‐rank test statistic, hazard ratio (HR), 95% confidence interval (CI), and p‐value. The significant HR greater than 1 indicates an increased risk of mortality for fish ≤ 32 cm compared to larger individuals.
**Supplementary Table 5.**Multiple logistic regression model summary: glm (m ~ TL + HT, family = binomial) of the survival probability of lab‐tagged Atlantic mackerel (Scomber scombrus). The odds ratio represents the multiplicative change in odds of fish survival for a one‐unit increase in the predictor variable (TL or handling time) while holding all other variables constant (Peng et al. 2002).
