# Supplementary material for: Surgical tagging of Atlantic mackerel (Scomber scombrus): electroanaesthesia and survival in captivity and the field
Source: J Fish Biol. 2026 Jan 22;108(4):1324–36. doi: 10.1111/jfb.70317 (PMC13193519; doi:10.1111/jfb.70317)
Supplement: Supplementary file 1 — Supplementary Figure 1.Relationships between wound condition and biological or handling metrics among surgically tagged Atlantic Mackerel (Scomber scombrus) that died within the 40‐day monitoring period. Boxplots show the distribution (from left to right) of days until death, handling time (sec), and total length (cm) for mackerel categorized by wound condition (a = fully healed, b = minor redness, c = severe redness) at time of recovery from laboratory tank. Only fish that died during the 40‐day post‐tagging monitoring period are included (n = 22). These exploratory comparisons were not tested statistically and were intended to visually assess trends. Supplementary Table 1.Metadata of lab‐tagged Atlantic mackerel (Scomber scombrus) including tag ID fate, date of death, handling time (seconds), total length (cm), and wound condition category (a = fully healed, b = minor redness, c = severe redness). The shaded boxes demonstrate the end of the 40‐day monitoring period. Supplementary Table 2.Metadata of field‐tagged Atlantic mackerel (Scomber scombrus) including fate: survived, censored, or mortality (not detected or deceased), date of death, date of last detection, and the receiver station of the last detection. Supplementary Table 3.Results of the Cox proportional hazards model assessing the effect of HT (sec) on survival time of lab‐tagged Atlantic mackerel (Scomber scombrus). The analysis includes the log‐rank test statistic, hazard ratio (HR), 95% confidence interval (CI), and p‐value. The significant HR less than 1 indicates a decreased risk of mortality for fish handled for ≤ 103 seconds compared to those handled for longer durations. Supplementary Table 4.Results of the Cox proportional hazards model assessing the effect of TL (cm) on survival time of lab‐tagged Atlantic mackerel (Scomber scombrus). The analysis includes the log‐rank test statistic, hazard ratio (HR), 95% confidence interval (CI), and p‐value. The significant HR greater than 1 indicates an inc [file JFB-108-1324-s001.pdf]

SUPPLEMENTAL

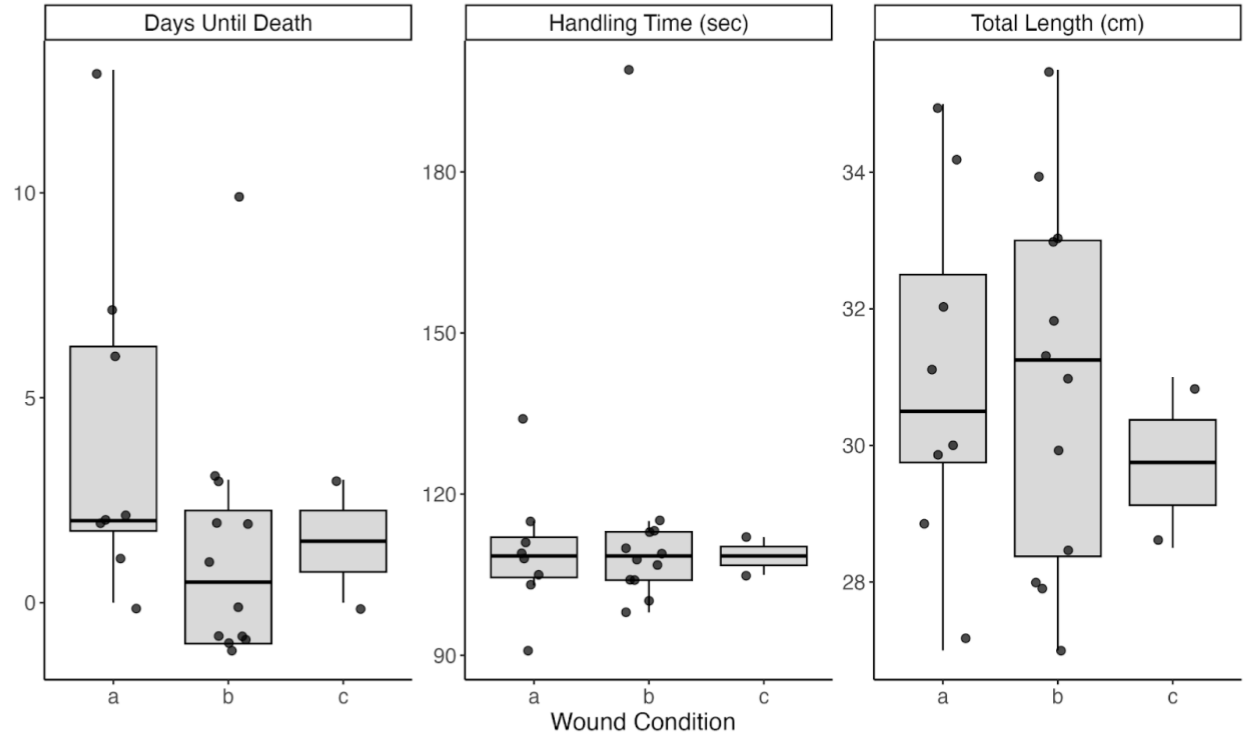

Figure 1. Relationships between wound condition and biological or handling metrics among surgically tagged Atlantic Mackerel (*Scomber scombrus*) that died within the 40-day monitoring period. Boxplots show the distribution (from left to right) of days until death, handling time (sec), and total length (cm) for mackerel categorized by wound condition (a = fully healed, b = minor redness, c = severe redness) at time of recovery from laboratory tank. Only fish that died during the 40-day post-tagging monitoring period are included (n = 22). These exploratory comparisons were not tested statistically and were intended to visually assess trends.

Table 1. Metadata of lab-tagged Atlantic mackerel (*Scomber* scombrus) including tag ID fate, date of death, handling time (seconds), total length (cm), and wound condition category (a = fully healed, b = minor redness, c = severe redness). The shaded boxes demonstrate the end of the 40-day monitoring period.

Tag ID	Fate	Date of Death (yyyy-mm-dd)	Handling Time (seconds)	Total Length (cm)	Wound Condition
25	Deceased	2023-11-10	100	34	b
18	Deceased	2023-11-10	107	27	b
10	Deceased	2023-11-10	113	33	b
30	Deceased	2023-11-10	109	33	b
37	Deceased	2023-11-10	113	28	b
17	Deceased	2023-11-11	105	31	c
8	Deceased	2023-11-11	100	34.5	c
7	Deceased	2023-11-11	110	28	b
0	Deceased	2023-11-12	105	30	a
21	Deceased	2023-11-12	104	28.5	b
44	Deceased	2023-11-13	111	29	a
47	Deceased	2023-11-13	134	35	a
23	Deceased	2023-11-13	199	32	b
22	Deceased	2023-11-13	98	35.5	b
55	Deceased	2023-11-13	103	31	a
11	Deceased	2023-11-14	112	28.5	c
50	Deceased	2023-11-14	115	31.5	b

Tag ID	Fate	Date of Death (yyyy-mm-dd)	Handling Time (seconds)	Total Length (cm)	Wound Condition
29	Deceased	2023-11-14	108	30	b
14	Deceased	2023-11-17	108	34	a
20	Deceased	2023-11-18	109	30	a
27	Deceased	2023-11-21	104	31	b
35	Deceased	2023-11-24	91	32	a
2023-12-21: End of the 40-day monitoring period					
5	Survived		92	36	b
48	Survived		93	36	b
56	Survived		35	94	a
1	Survived		93	35	a
9	Survived		121	36	a
39	Survived		91	35	b
52	Survived		101	33	a
4	Survived		105	33	a
3	Survived		101	30	b
36	Survived		98	32	b
46	Survived		49	29	a
38	Survived		97	28	a
24	Survived		46	34	b
51	Survived		91	33	a
32	Survived		94	36	b

Tag ID	Fate	Date of Death (yyyy-mm-dd)	Handling Time (seconds)	Total Length (cm)	Wound Condition
49	Survived		89	39	b
28	Survived		97	33	a
16	Survived		102	31	a
15	Survived		115	27	a

Table 2. Metadata of field-tagged Atlantic mackerel (*Scomber scombrus*) including fate: survived, censored, or mortality (not detected or deceased), date of death, date of last detection, and the receiver station of the last detection.

Tag ID	Fate	Date of Death (yyyy-mm-dd)	Date of Last Detection	Receiver Station
A69-1303-8948	Censored	NA	2024-09-10	9
A69-1303-8958	Survived	NA	2024-07-29	9
A69-1303-8959	Survived	NA	2024-08-17	10
A69-1303-8960	Survived	NA	2024-09-01	13
A69-1303-8961	Survived	NA	2024-08-19	9
A69-1303-8962	Survived	NA	2024-07-27	8
A69-1303-8963	Survived	NA	2024-08-12	10
A69-1303-8978	Survived	NA	2024-08-13	10
A69-1303-8979	Not Detected	2024-07-24	NA	NA
A69-1303-8980	Deceased	2024-08-08	2024-08-18	10
A69-1303-8981	Survived	NA	2024-07-27	8
A69-1303-8982	Not Detected	2024-07-24	NA	NA
A69-1303-8983	Survived	NA	2024-07-27	9
A69-1303-9008	Survived	NA	2024-09-02	13
A69-1303-9009	Survived	NA	2024-07-25	10
A69-1303-9010	Not Detected	2024-07-24	NA	NA
A69-1303-9011	Survived	NA	2024-08-27	10
A69-1303-9012	Censored	NA	2024-07-26	10

Tag ID	Fate	Date of Death (yyyy-mm-dd)	Date of Last Detection	Receiver Station
A69-1303-9013	Censored	NA	2024-07-27	13
A69-1303-9064	Survived	NA	2024-08-13	13
A69-1303-9065	Survived	NA	2024-09-14	10
A69-1303-9066	Survived	NA	2024-08-12	12
A69-1303-9067	Survived	NA	2024-08-11	10
A69-1303-9068	Survived	NA	2024-07-25	10
A69-1303-9069	Survived	NA	2024-07-29	8
A69-1303-9070	Survived	NA	2024-08-12	9
A69-1303-9071	Censored	NA	2024-07-28	8
A69-1303-9072	Survived	NA	2024-09-12	10
A69-1303-9073	Survived	NA	2024-08-06	7
A69-1303-9084	Survived	NA	2024-08-29	10
A69-1303-9085	Survived	NA	2024-08-21	10
A69-1303-9086	Survived	NA	2024-08-13	13
A69-1303-9087	Censored	NA	2024-08-06	16
A69-1303-9094	Survived	NA	2024-08-27	10
A69-1303-9095	Survived	NA	2024-08-26	8
A69-1303-9096	Censored	NA	2024-08-24	10

Tag ID	Fate	Date of Death (yyyy-mm-dd)	Date of Last Detection	Receiver Station
A69-1303-9097	Survived	NA	2024-09-05	10
A69-1303-9102	Survived	NA	2024-07-27	8
A69-1303-9103	Censored	NA	2024-07-27	10
A69-1303-9104	Censored	NA	2024-08-16	10
A69-1303-9105	Censored	NA	2024-08-13	16
A69-1303-9106	Survived	NA	2024-09-16	10
A69-1303-9107	Survived	NA	2024-08-29	10
A69-1303-9232	Survived	NA	2024-07-28	10
A69-1303-9233	Survived	NA	2024-08-14	10
A69-1303-9236	Survived	NA	2024-09-15	10
A69-1303-9237	Censored	NA	2024-07-26	12
A69-1303-9242	Censored	NA	2024-11-07	10
A69-1303-9243	Survived	NA	2024-08-06	8
A69-1303-9303	Survived	NA	2024-08-17	10

Table 3. Results of the Cox proportional hazards model assessing the effect of HT (sec) on survival time of lab-tagged Atlantic mackerel (*Scomber scombrus*). The analysis includes the log-rank test statistic, hazard ratio (HR), 95% confidence interval (CI), and p-value. The significant HR less than 1 indicates a decreased risk of mortality for fish handled for ≤ 103 seconds compared to those handled for longer durations.

Predictor	Coefficient (β)	Hazard Ratio	95% CI (Lower - Upper)	p-value	Test Statistics
HT ≤ 103sec	-2.3024	0.100	0.0327 - 0.3059	5.43e-05 ***	Log-rank χ^2 = 23.1, p = 2e-06

Signif. codes: 0 ‘***’ 0.001 ‘**’ 0.01 ‘*’ 0.05 ‘.’ 0.1 ‘ ’ 1

Table 4. Results of the Cox proportional hazards model assessing the effect of TL (cm) on survival time of lab-tagged Atlantic mackerel (*Scomber scombrus*). The analysis includes the log-rank test statistic, hazard ratio (HR), 95% confidence interval (CI), and p-value. The significant HR greater than 1 indicates an increased risk of mortality for fish ≤ 32 cm compared to larger individuals.

Predictor	Coefficient (β)	Hazard Ratio	95% CI (Lower - Upper)	p-value	Test Statistics
TL ≤ 32cm	1.2161	3.3741	1.31 - 8.69	0.012 *	Log-rank χ^2 = 7.12, p = 0.008

Signif. codes: 0 ‘***’ 0.001 ‘**’ 0.01 ‘*’ 0.05 ‘.’ 0.1 ‘ ’ 1

Table 5. Multiple logistic regression model summary: glm(m ~ TL + HT, family = binomial) of the survival probability of lab-tagged Atlantic mackerel (*Scomber scombrus*). The odds ratio represents the multiplicative change in odds of fish survival for a one-unit increase in the predictor variable (TL or handling time) while holding all other variables constant (Peng et al. 2002).

	Odds Ratio	Std. Err.	z value	P > |z|	[95% Conf.	Interval]
(Intercept)	16.58608	9.49677	0.296	0.7674	-13.94031	24.43458
TL	1.44387	0.19357	1.898	0.0577	1.00318	2.20726
HT	0.86574	0.06157	-2.341	0.0192 *	7.46687	9.52088

Signif. codes: 0 ‘***’ 0.001 ‘**’ 0.01 ‘*’ 0.05 ‘.’ 0.1 ‘ ’ 1
